# Correlation of HER2 With p53 and p63 in Urothelial Bladder Carcinoma

**DOI:** 10.7759/cureus.38018

**Published:** 2023-04-23

**Authors:** Divya Madhuri Ponnaboina, Sushmitha Perumandal, Sreelakshmi I

**Affiliations:** 1 Pathology, Osmania Medical College, Hyderabad, IND

**Keywords:** her2 neu protein, p63 oncogene, p53 oncogene, urothelial carcinoma, bladder carcinoma

## Abstract

Urothelial carcinomas account for the majority of all primary bladder cancers, making bladder cancer the second most frequent genitourinary malignancy after prostate cancer. Bladder cancer risk rises with age and most of them return after resection due to their multifocal distribution, and they often develop in superficial locations. Like many other cancers, bladder carcinoma is associated with a few tumor markers that have been evaluated in the past. They include p53, p63, and HER2. This study was conducted on 88 patients suspected of urinary bladder carcinoma. This prospective study was done at the Department of Pathology, Osmania General Hospital, Hyderabad from August 2017 to July 2019. Of the 88 patients, 76 were diagnosed with bladder carcinoma and the remaining 12 were non-neoplastic. The primary neoplastic lesions of the urinary bladder were predominantly seen in patients older than 40 years and were found to be statistically significant (p<0.01). Of the 34 cases of high-grade papillary urothelial carcinoma (PUC), 26 (76.47%) were males, eight cases (23.53%) were females, while among the 25 cases of low-grade PUC, 20 cases (80%) were males, and five cases (20%) were females. In seven cases of squamous cell carcinoma, six cases (85.71%) were males and only one case (14.29%) was female. Of the two cases of adenocarcinoma, male and female gender accounted for one case each (50%). The two cases of papillary urothelial neoplasm of low malignant potential were males in the study. On the whole, the primary urinary bladder lesions are more predominant in the males (77.63%) than the females (22.37%). Overexpression of p53 is negatively connected to p63 expression, and HER2 and p53 were strongly associated with high tumor grade in urothelial carcinoma.

## Introduction

Urothelial carcinomas account for 90% of all primary bladder cancers, making bladder cancer the second most frequent genitourinary malignancy after prostate cancer. Almost 4% of all urological lesions are urinary bladder tumors. After colorectal, lung, and prostate cancer, bladder cancer is the fourth most frequent cancer among males in the United States. After breast, lung, colorectal, uterine, non-lymphoma, Hodgkin's thyroid, and ovary, it is the eighth most prevalent cancer in women [1].

Bladder cancer risk rises with age, with the median age of diagnosis at 65 years, and is a rarity before age 40. Seventy percent of urothelial carcinomas return after resection due to their multifocal distribution, and they often develop in superficial locations. In the majority of patients, there is a delay in the patient's arrival, and the tumor has advanced past the point at which curative therapy is no longer an option. The high likelihood of recurrence following a successful resection is also a result of the inability to identify the carcinogenic agent, which is frequently unknown [2,3]. HER2, p53, and p63 are closely associated with bladder carcinoma. This study has been undertaken to correlate the expression of HER2 with p53 and p63 and their correlation with clinicopathological characteristics.

## Materials and methods

The present study is a prospective study conducted at The Department of Pathology, Osmania General Hospital, Hyderabad, Telangana over two years from August 2017 to July 2019. Out of the total 88 cases, after excluding autolyzed specimens and benign lesions, 76 transurethral resection of bladder tissue (TURBT) biopsies were included in the study for tumor marker analysis. After taking informed consent, all the confirmed cases of urinary bladder lesions were examined for cystoscopic and clinical findings, and relevant history was taken. Biopsy specimens from TURBT received in 10% formalin were processed as per routine histopathological techniques followed by H&E staining and then an immunohistochemistry panel of HER2, p53, and p63. The slides were studied and classified according to the WHO histological classification of tumors of the urinary tract in 2022. Immunohistochemical staining was done using PEROXIDASE - ANTIPEROXIDASE method according to the protocol described by Biogenics. Primary antibodies for HER2 are DAKO (Monoclonal Mouse Anti-Human Ks 20.8), for p53 are Biogenex (Monoclonal Mouse Anti-Human D07), and for P63 are Biogenex (Monoclonal Mouse Anti-Human MIB-1). The WHO/ISUP Classification was used to grade the tumors into urothelial papilloma, papillary urothelial neoplasm of low malignant potential (PUNLMP), and low- and high-grade urothelial (transitional cell) carcinomas (TCCs). Pathological staging of the urothelial cancers was done according to the TNM (tumor, node, metastasis) system and data were recorded as pT0: tumor limited to the mucosa, pT1: invasion of lamina propria (subepithelial connective tissue), and pT2: an invasion of muscle. This study was conducted according to the guidelines laid by the Declaration of Helsinki. Statistical analysis was done using Microsoft Excel 2013. Mann-Whitney U test was employed to test the significance. Institutional ethics committee clearance was obtained from the Institutional Ethics Committee, Osmania Medical College, Hyderabad.

## Results

During these 24 months, a total of 88 cases were included in the study, 76 cases were neoplastic lesions accounting for 66.88%, whereas 12 cases, about 13.63%, were non-neoplastic lesions. The youngest patient in this study was 25 years old and the oldest patient was 85 years old. The mean age was 55.6 years. Of these 88 cases, 42 cases (47.72%) were in the age group of 51-70 years. Out of 88 patients, a male-to-female ratio of 3:1 was observed with 67 males (76.13%) and 21 females (23.86%). Among these 67 male patients, most of the patients showed neoplastic lesions accounting for 59 cases (88.05%), and the remaining eight cases (11.94%) were non-neoplastic lesions. Among the 21 female patients, 17 cases (80.95%) were neoplastic and four cases (19.04%) were non-neoplastic lesions. All the types of neoplastic lesions predominated in the age group of 51-70 years. The primary neoplastic lesions of the urinary bladder were predominantly seen in patients older than 40 years and were found to be statistically significant (p<0.01). There were 25 cases of low-grade papillary urothelial carcinoma (PUC), with 20 cases (80%) being male and five cases (20%) being female. Of the 34 cases of high-grade PUC, 26 (76.47%) were male and eight (23.53%) were female. Each gender contributed one case (50%) to a total of two adenocarcinoma cases. In the study, there were two male cases with PUNLMP. In general, males (77.63%) have more primary urinary bladder lesions than females (22.37%). Of the 34 cases of high-grade PUC, 26 (76.47%) were males, eight cases (23.53%) were females, while among the 25 cases of low-grade PUC, 20 cases (80%) were males and five cases (20%) were females. In seven cases of SCC, six cases (85.71%) were males and only one case (14.29%) was female. In two cases of adenocarcinoma, male and female gender accounted for one case each (50%). The two cases of PUNLMP were males in the study. On the whole, the primary urinary bladder lesions were more predominant in the males (77.63%) than the females (22.37%). The most common presenting symptom among the patients included in this study was hematuria in 70 cases (79.54%) followed by abdominal pain in 58 cases (65.9%), increased frequency of micturition in 42 cases (47.72%), and urinary urgency in 38 cases (43.18%); four other symptoms like mass per abdomen, dysuria, dribbling of urine, and polyuria were seen less frequently in 24 cases (23.14%). Patients with >20% tumor cell staining were considered immunohistochemistry positive for the concerned marker. The HER2, p53, and p63 positivity are mentioned in Tables 1-5.

**Table 1:** IHC of PUNLMP IHC, immunohistochemistry; PUNLMP, papillary urothelial neoplasm of low malignant potential.

IHC-PUNLMP			
N=02	+1	+2	+3
p53	1	1	0
p63	0	1	1
HER2	2	0	0

**Table 2:** Immunohistochemistry of LGPUC LGPUC, low-grade papillary urothelial carcinoma.

N=25	+1	+2	+3
p53	10	12	3
p63	2	8	15
HER2	8	14	3

**Table 3:** Immunohistochemistry of HGPUC HGPUC, high-grade papillary urothelial carcinoma.

N=34	+1	+2	+3
p53	1	5	28
p63	30	3	1
HER2	1	7	26

**Table 4:** Immunohistochemistry of HGPUC with squamous differentiation HGPUC, high-grade papillary urothelial carcinoma.

N=6	+1	+2	+3
p53	1	3	2
p63	4	2	0
HER2	0	2	4

**Table 5:** Immunohistochemistry of invasive PUC with squamous differentiation PUC, papillary urothelial carcinoma.

N=7	+1	+2	+3
p53	1	5	1
p63	5	1	1
HER2	1	1	5

## Discussion

Bladder cancer is the seventh most prevalent malignancy overall, with an estimated 2,60,000 new men diagnosed each year. According to other research, the incidence of bladder cancer climbs gradually, peaking between the sixth and seventh decade. Males seem to experience it more frequently than females do. The results of our investigation also support these conclusions. This data consequently implies that while estrogen hormones are protective, some androgenic hormones induce oncogenesis. It is recognized that dietary practices, tobacco use, environmental variables, occupational factors, and lifestyle factors all affect the development of urothelial carcinoma [4,5]. This study consists of 88 cases of urinary bladder lesions; among these, 12 cases were inflammatory lesions and the remaining 76 cases were neoplastic lesions. The age of presentation of bladder cancers is predominantly in the age group of 51-70 years with a mean age of presentation being 62 years similar to other studies [1]. The male-to-female ratio in this study is predominately male (67 cases), with a male-to-female ratio of roughly 3.5:1 (21 cases). Our study's sex ratio is larger than that of Laishram et al., but lower than that of many other studies [1]. We found 52 smokers (59.1%). Several non-smokers reported chewing tobacco. Men were 85%, habitual smokers. These were similar to the study by Biswas et al. with 75% smokers [4]. A causal relationship between bladder cancer and occupational exposure is well established and concurrent smoking increases the risk to about 90-fold. There are limited studies on occupation and bladder cancers in our country. In the present study, the majority of patients are farmers 25 cases (28.4%) and laborers 24 cases (27.2%). In the studies done by Biswas et al and Chinnasamy et al showed increased risk among laborers (50%) and industrial workers, including the leather and textile industries, hair dye handlers, and shoemakers [4,6]. Of all the 76 epithelial-origin bladder cancers, urothelial (transitional cell) tumors constituted around 67 cases (88.15%). The most common bladder neoplasm in the present study is PUC, seen in 67 cases (88.15%). Similar data (82.60%) were published by Shah et al. [7].

In this study, SCC accounted for seven cases (9.21%), which is similar to the study by Shah et al. [7], which showed two cases (8.69%) of SCC. TCC is diagnosed in 59 cases in the present study. Of these, 54 cases (90%) are superficial or in the early stage without muscle invasion, while five (10%) cases show muscle invasion. In the study done by Shah et al., muscle invasion was seen in 31.25% [7]. Not including the muscle layer in the biopsy specimens may lead to the under-staging of tumors in many patients. The importance of including the muscle layer in the biopsy specimens needs to be emphasized for the efficient staging of tumors. In this study, two cases of PUNLMP show weak nuclear positivity in one case (50%) and moderate nuclear positivity in another case (50%). Expression of p63 in PUNLMP is consistent with studies done by Rajcani et al. [8] and Eva Comparet et al. [9], i.e. moderate and strong nuclear positivity in one case each. Expression of HER2 in both cases of PUNLMP is weak membrane positivity, which is similar to the study done by Lim SD et al. [10].

Out of 25 cases of LGPUC, 12 (48%) and three (12%) cases show moderate and strong nuclear positivity for p53, which is consistent with the study by Raheem et al. [11]. Expression of p63 in eight cases (32%) is moderate nuclear positivity and in 15 cases (60%) was strong nuclear positivity which is similar to a study done by Ali koyuncuer [12]. In this study, 34 cases were included under HGPUC, out of which 28 cases (82.35%) show strong nuclear positivity for p53, which is consistent with the study done by Raheem et al. [11]. HER2 plays a critical role in cell proliferation and tumorigenesis; it has been widely studied in breast cancer as a poor prognostic factor and therapeutic target. Strong membrane positivity for HER2 was seen in 26 cases (76.47%) out of 34 cases of HGPUC. This finding is similar to a study done by Mejri et al. [13]. Our study showed a correlation between HER2 overexpression and tumor grade with a significant p-value similar to Charfi et al. [5]. In the present study, the expression of p53 in various grades of urothelial carcinomas was analyzed and it demonstrated a significant association with high tumor grade, which is consistent with the study by Qamar et al. [14]. With the above results and observations, this study intended to evaluate the expression of p53, p63, and HER2 in urothelial carcinoma and analyze its association with tumor grade. In the present study, there was a significant relationship between overexpression of p53, p63, and HER2 with tumor grade, and these results were in line with others. From our results, the three markers in the study were important prognostically.

## Conclusions

The most prevalent bladder tumor in our analysis was high-grade TCC. Our analysis found several high-grade TCCs without muscle invasion. Overexpression of p53 is negatively connected to p63 expression and HER2 and p53 were strongly associated with high tumor grade in urothelial carcinoma.
